# Dental caries and anthropometrics of children living in an informal floating Amazonian community: a cross-sectional pilot study

**DOI:** 10.1590/0103-6440202204310

**Published:** 2022-03-07

**Authors:** Ana Lucia Seminario, Elizabeth Alpert, Eduardo Bernabé, Jennifer Liu, Leann Andrews, Jorge A. Alarcón, Mauro Milko Echevarría Chong, Joseph Zunt

**Affiliations:** 1 Department of Pediatric Dentistry, School of Dentistry, Department of Global Health, School of Public Health, University of Washington, United States of America; 2 School of Dentistry, Universidad Peruana Cayetano Heredia, Peru; 3 Department of Oral Health Policy and Epidemiology, Harvard School of Dental Medicine, United States of America; 4 Division of Population and Patient Health, King's College London, United Kingdom; 5 Department of Epidemiology, School of Public Health, University of Washington, United States of America; 6 Centro de Investigaciones Tecnológicas, Biomédicas y Medioambientales (CITBM), Peru; 7 College of Built Environment, University of Washington, United States of America; 8 Department of Global Health, University of Washington, United States of America; 9 Department of Dentistry, School of Dentistry, Universidad Nacional de la Amazonía Peruana, Peru; 10 Department of Global Health, School of Public Health, Department of Neurology, School of Medicine, University of Washington, United States of America

**Keywords:** Oral health, dental caries, pediatrics, growth, floating communities

## Abstract

Aims: Increasing evidence supports a relationship between poor oral health and growth in children. Our objective was to assess the association between the presence of dental caries and anthropometric measurements of children residing in Claverito, a floating slum community in the Peruvian Amazon. Methods: For this cross-sectional study, presence of caries was assessed using dmft/DMFT (decayed, missing, filled teeth) scores and the SiC Index (mean dmft/DMFT of one-third of the study group with the highest caries score). Anthropometric categories for age-sex-specific z-scores for height and weight were calculated based on WHO standardized procedures and definitions. The association between SiC (measured by dmft/DMFT) and anthropometric measures was estimated using unadjusted and adjusted multivariable linear regression models. Critical value was established at 5%. Results: Our study population consisted of 67 children between the ages of 1 and 18 years old. Mean age was 9.5 years old (SD: 4.5), and the majority were female (52.2%). Almost all had dental caries (97.0%) and the mean dmft/DMFT score was 7.2 (SD: 4.7). The SiC Index of this population was 9.0. After adjusting for confounding variables, participants who had permanent dentition with the highest dmft/DMFT levels had statistically significant decreased height-for-age z-scores (HAZ) (p=0.04). Conclusions: We found an inverse linear association between SiC Index and height-for-age z-scores (HAZ) among children living in poverty in a floating Amazonian community in Peru. Children from under-resourced communities, like floating slums, are at high risk for oral disease possibly negatively impacting their growth and development.

## Resumo

Objetivos: Há cada vez mais evidências apoiando uma relação entre a saúde bucal precária e o crescimento das crianças. Nosso objetivo foi avaliar a associação entre a presença de cárie dentária e medidas antropométricas de crianças residentes em Claverito, uma comunidade flutuante de favelas na Amazônia peruana. Métodos: Para este estudo transversal, a presença de cárie foi avaliada utilizando os escores dmft/DMFT (dentes cariados, ausentes, preenchidos) e o Índice SiC (média dmft/DMFT de um terço do grupo de estudo com a maior pontuação de cárie). As categorias antropométricas para pontuações z específicas de idade e sexo para altura e peso foram calculadas com base nos procedimentos e definições padronizados da OMS. A associação entre SiC (medido por dmft/DMFT) e medidas antropométricas foi estimada usando modelos de regressão linear multivariável não ajustados e ajustados. O valor crítico foi estabelecido em 5%. Resultados: A população do estudo consistia de 67 crianças entre 1 e 18 anos de idade. A idade média era de 9,5 anos (DP: 4,5), e a maioria era do sexo feminino (52,2%). Quase todas tinham cárie dentária (97,0%) e a média da pontuação dmft/DMFT foi de 7,2 (DP: 4,7). O índice SiC desta população era de 9,0. Após ajuste para variáveis confusas, os participantes que tinham dentição permanente com os níveis mais altos de dmft/DMFT tinham diminuído estatisticamente a pontuação z de altura por idade (HAZ) (p=0,04). Conclusões: Encontramos uma associação linear inversa entre o Índice SiC e as pontuações z de altura por idade (HAZ) entre crianças que vivem na pobreza em uma comunidade amazônica flutuante no Peru. Crianças de comunidades com poucos recursos, como favelas flutuantes, correm alto risco de contrair doenças orais, possivelmente impactando negativamente seu crescimento e desenvolvimento.

## Introduction

### Background/rationale

Floating communities living in poverty are found globally. They face varying challenges based upon factors including proximity to urban areas, accessibility, geographic characteristics, environmental conditions, and national and local policies.^1^ The United Nation’s (UN) Sustainable Development Goals (SDGs) seek to improve quality of life for these communities through objectives including eliminating poverty, facilitating clean water and sanitation, promoting decent work and economic growth, and improving sustainability.^2^

The World Health Organization (WHO) notes that children’s health is especially affected by slum living conditions.^3^ Socioeconomic inequality leading to malnutrition can be measured by indicators including stunting, wasting, underweight and overweight habitus,^4^ which are quantified in terms of deviation from the WHO Child Growth Standards median. Stunting, a decrease in linear growth, is defined as height-for-age less than two standard deviations from the median. Wasting, reduced body tissue mass and unique physiological abnormalities, is quantified as weight-for-height less than two standard deviations from the median. Underweight, a composite indicator representing stunting and/or wasting, is defined as a weight-for-age less than two standard deviations from the median. Lastly, overweight, higher than a healthy weight for a given height, is quantified by a weight-for-height greater than two standard deviations from the median.^5^

Dental caries (tooth decay) is the most common chronic disease affecting children^6^ and negatively impacts anthropometrics^7^ and well-being.^6^ Previous studies have demonstrated associations between high levels of untreated caries and poorer growth.^7^ ^),(^ ^8^

The effects of caries on anthropometrics are attributed to multiple proposed mechanisms, both direct and indirect. Directly, caries and associated pain can lead to undernutrition and ultimately limit growth. Additionally, severe dental caries can result in indirect effects on growth by triggering metabolic and endocrine responses. These responses include caloric wasting and impaired nutrient absorption, as well as the child’s decreased ability to sleep, which decreases growth hormone secretion. Furthermore, infection caused by caries can negatively impact the immune system. Individually and collectively, these proposed mechanisms explain how severe dental caries can ultimately lead to decreased anthropometric measures of height and weight.^7^

As an upper-middle-income country, Peru exhibited rapid economic growth over the last decade. Yet over 20% of the population continue to live below the national poverty line. This uneven wealth distribution is consistent with other Latin American countries, which exhibit the highest socioeconomic inequality in the world.^9^ The floating community of Claverito, Iquitos, is located in northeastern Peru on the Itaya River, a tributary of the Amazon. Claverito is comprised of over 200 migrant people living in approximately 50 households.^10^ 89% of households live in poverty, with income less than 305 soles (93 USD) per person per month, or extreme poverty, with income less than 175 soles (53 USD) per person per month.^11^

InterACTION Labs is a transdisciplinary action research program in which a research team is collaborating with the Claverito community on health research and interventions to improve environmental conditions in this floating informal slum community.^10^ In 2017, the University of Washington (UW) Population Health Initiative, the Centro de Investigaciones Tecnológicas, Biomédicas y Medio Ambientales (CITBM) and 100,000 Strong in the Americas awarded InterACTION Labs a pilot study to understand and address health disparities in Claverito.

## Objective

The objective of this study was to examine associations between oral health and the anthropometrics of children residing in the floating urban slum community of Claverito. We hypothesized that children with the most oral disease would have higher levels of stunting, wasting, and underweight status than children with lower levels of disease. 

This pilot study was intended to enlargen the body of literature on how oral health affects the growth of children living in floating slum communities and facing poverty. This study also explores the use of the Significant Caries Index (SiC Index) metric, mean dmft/DMFT of one-third of the study group with the highest caries score. In underserved areas with extremely high prevalence of caries, this is a more sensitive disease indicator than dmft/DMFT scores and reflects disease severity. Previous studies have not examined this metric in the context of the impact of dental disease on childhood growth.

## Methods

### Study design

This cross-sectional study was designed to compare measurements of height and weight of children living in Claverito with the mean number of decayed, missed, and filled teeth due to caries (dmft/DMFT), adjusting that association for possible confounding factors. This study was approved by the University of Washington Ethics Committee (#STUDY00000022) and by the Instituto de Medicina Tropical, Daniel Alcides Carrion, at the Universidad Nacional Mayor de San Marcos in Peru (#CIEI-2018-004). The STROBE (Strengthening the Reporting of Observational Studies in Epidemiology) guidelines for reporting observational studies were followed.^12^

### Setting

InterACTION Labs is a transdisciplinary built environment-community health program focusing on the informal floating community of Claverito, Iquitos, Peru. Addressing both the built and natural environments, the InterACTION Labs project was designed to improve living and health conditions in impoverished communities through participatory design, implementation, and assessment.^10^ The team includes representatives from the University of Washington, Universidad Nacional Mayor de San Marcos Centro de Investigaciones Tecnológicas, Biomédicas y Medio Ambientales and the Universidad Nacional de la Amazonia Peruana from departments including Public Health, Global Health, Epidemiology, Nursing, Dentistry, Neurology, Landscape Architecture, Environmental and Occupational Health, and Environmental Engineering.

Recruitment for this study began in 2018, and data collection was performed by a multidisciplinary research team over two days in February 2018. Informed consent/assent was obtained, and demographic information was collected from pediatric participants and their parents or caregivers. Data collection was performed in the participant’s house. If a member of the family was not present on the first day of data collection, the team returned to reattempt data collection on the second day.

### Participants

At the time of the study, the community included 270 members residing in 50 households, ranging in age from 1 month to 82 years. All were invited to participate, and 211 participated in at least one aspect of the InterACTION Labs project. Eligible subjects for this study had at least 1 tooth, were 18 years of age or younger, had physical exam findings (height and weight), were available for oral examinations, and had signed informed consent from their parents or caregivers. Of the 211 participating residents, 109 (51.7%) were eligible for this study. Of these 109 children, 67 were present and consented for participation.

### Variables

- Outcomes
  - Anthropometrics: height, weight, z-scores of height-for-age (HAZ), weight-for-age (WAZ), weight-for-height, and body mass index for age (ZBMI)
- Exposures
  - Dental factors: mean number of decayed, missing, and filled teeth due to caries (dmft/DMFT) score, caries experience (sum of dmft and DMFT), and Significant Caries Index (SiC Index) (mean dmft/DMFT of one-third of the study group with the highest caries score)
- Effect modifiers
  - Dental factors: type of teeth (primary, mixed and permanent dentition)
- Potential confounders
  - Demographics: sex, age
  - Health status: self-reported diagnosed health conditions (if diagnosed by a doctor)
  - Food and nutrition: Household Food Insecurity Access Scale (HFIAS)
- Data sources/measurement
- Anthropometrics: height, weight, z-scores of height-for-age (HAZ), weight-for-age (WAZ), weight-for-height, and body mass index for age (ZBMI). WHO standardized procedures were used to estimate HAZ, WAZ, and ZBMI scores. Indicators of childhood malnutrition (stunting, underweight, and wasting) were determined based on the WHO Child Growth Standards.^5^ Stunting refers to height-for-age less than 2 SDs from the median, underweight was defined by weight-for-age less than 2 SDs from the median, and wasting was defined by weight-for-height less than 2 SDs from the median.^5^
- Dental factors: type of teeth (primary, mixed and permanent dentition), mean number of decayed, missing, and filled teeth due to caries (dmft/DMFT) score, caries experience (sum of dmft and DMFT), and Significant Caries Index (SiC Index) (mean dmft/DMFT of one-third of the study group with the highest caries score). Dentition status was recorded for participants using the standardized WHO Oral Assessment form^13^ by a trained team of Peruvian and Spanish-speaking public health dentists. Data was collected using WHO criteria.^13^
- Demographics: sex, age. Demographic information was collected from pediatric participants and their parents or caregivers.
- Health status: self-reported diagnosed health conditions (if diagnosed by a doctor). Data was collected via a survey with prompts about conditions in categories of diarrhea and stomach problems, fever, respiratory, skin conditions, vision, hearing, heart, muscular system, neurological, endocrinology, allergies, infectious diseases, diabetes, cancer, and environmental intoxication.
- Food and nutrition: Household Food Insecurity Access Scale (HFIAS). The HFIAS is a 9-question survey validated by the USAID Food and Nutrition Technical Assistance (FANTA) with questions about food anxiety, food type and variety, meal size, meal quantity and missed meals.^14^

### Bias

To reduce bias on sampling representing the entire Claverito population, all 270 community members were invited to participate, and 211 individuals participated in at least one aspect of the InterACTION Labs project. To increase participation, data collection occurred over multiple days. To minimize bias occurring from data collection by several examiners, training and calibration sessions were conducted.

### Study size

Of the 211 participating Claverito residents, 109 were eligible for this study. Of these 109 children, 67 were present and consented for participation.

### Quantitative variables

Regarding data management, survey variables were recorded on paper forms and entered into and managed using REDCap (Research Electronic Data Capture) tools hosted at the University of Washington. REDCap is a secure web application designed to compile and analyze surveys and databases.^15^

Anthropometric categories for age-sex-specific z-scores for height and weight were calculated based on WHO standardized procedures and definitions.^5^ ^),(^ ^16^ ^),(^ ^17^ Because WHO does not provide z-scores for weight in children beyond age 10, z-scores for BMI (ZBMI) were calculated based on WHO standardized procedures for purposes of analyses.^17^ Distributions of the sample population were assessed and frequency and percentage of study variables calculated.

Participants were stratified by SiC and non-SiC. For our sample, a mean caries experience score greater than or equal to 9.0 placed participants into the one-third of the study group with the highest caries score.

### Statistical methods

Unadjusted and adjusted multivariable linear regression models were used to estimate the association between dmft/DMFT and anthropometric measures (HAZ and ZBMI), and similar models were also used to estimate the association between SiC and anthropometric measures (HAZ and ZBMI). All models were adjusted for sex, age, presence of diagnosed health conditions, and HFIAS categories. To examine if dentition stage (primary, mixed, permanent) modified the associations, interaction analyses and stratum-specific estimates were calculated. Statistical significance was set at 5%, and R software version 3.6.0 was utilized for analyses.

## Results

### Participants

At the time of our study, Claverito had 109 residents ages 18 and under who were potentially eligibility for the study. Of these 109, 67 (61.5%) were examined for elgibility, confirmed eligible, present for data collection, and agreed to participate (Table 1). Anthropometric data was missing for 14 participants due to inability to tolerate measurements.

### Descriptive data

The majority were female (52.2%) and mean age was 9.5 (SD: 4.5) while median was 9.0. 70.2% of the study population reported no diagnosed health conditions, and 65.7% were severely food insecure. Almost half (44.8%) were in mixed dentition stage while 32.8% and 22.4% were in permanent and primary dentition phases, respectively. While half of the study population had normal weight (37, 55.2%), 13 were stunted (19.4%), and 1 exhibited wasting (1.5%). There were no participants considered overweight.

### Outcome data

Prevalence of dental caries was 97.0%. Mean dmft/DMFT was 7.2 (SD: 4.7) and median was 7.0 (IQR: 3.0, 10.5). The SiC Index of this population was 9.0.

### Main results

Linear regression models were used to estimate the association between caries experience (sum of dmft and DMFT) with continuous HAZ and continuous ZBMI. Table 2 demonstrates that for our study

population, these associations were not significant. That was true regardless of dentition status and after adjusting for confounders.

**Table 1:** Demographic variables by dental caries status

Variables	N (%)
Demográficas	
Sex	
Female	35 (52.24%)
Male	32 (47.76%)
Age	
≤ 5 years	14 (20.90%)
6 years - 12 years	33 (49.25%)
13 years - 18 years	20 (29.85%)
Health status	
Health Conditions	
No diagnosed health conditions	47 (70.15%)
Diagnosed health conditions	17 (25.37%)
Missing data	3 (4.48%)
Food and nutrition	
Household Food Insecurity Access Scale (HFIAS) Category	
Food secure	6 (8.96%)
Mildly food insecure	1 (1.49%)
Moderately food insecure	13 (19.40%)
Severely food insecure	44 (65.67%)
Missing data	3 (4.48%)
Dental factors	
Type of dentition	
Primary	15 (22.39%)
Mixed	30 (44.78%)
Permanent	22 (32.84%)
Caries Present	65 (97.01%)
Antropométricas*	
Normal weight	37 (55.22%)
Underweight	2 (2.99%)
Stunting^**^	13 (19.40%)
Wasting^***^	1 (1.49%)
Missing data	14 (20.90%)

*No participants in “Overweight” category; arm circumference-for-age and height-for-weight not included due to low sample size

**Stunting is defined as height-for-age less than two standard deviations from the median.

***Wasting is quantified as weight-for-height less than two standard deviations from the median.

**Table 2:** Association between caries experience and anthropometric measures (all dentition and interaction by dentition stage)*

	Height for age z-scores (HAZ) (95% CI)		Body mass index z-scores (ZBMI) (95% CI)	
	Unadjusted (N = 53)	Adjusted (N = 52)	Unadjusted (N = 53)	Adjusted (N = 52)
All Dentition	-0.03 (-0.10, 0.04) p-value = 0.37	-0.04 (-0.11, 0.04) p-value = 0.31	-0.03 (-0.08, 0.02) p-value = 0.28	-0.03 (-0.08, 0.02) p-value = 0.20
Primary Dentition	0.09 (-0.08, 0.27) Stratum-specific p-value = 0.30	0.09 (-0.10, 0.28) Stratum-specific p-value = 0.35	-0.07 (-0.19, 0.05) Stratum-specific p-value = 0.23	-0.12 (-0.24, 0.001) Stratum-specific p-value = 0.05
Mixed Dentition	-0.01 (-0.07, 0.04) Stratum-specific p-value = 0.58	0.004 (-0.06, 0.07) Stratum-specific p-value = 0.91	0.02 (-0.06, 0.10) Stratum-specific p-value = 0.67	-0.001 (-0.08, 0.08) Stratum-specific p-value = 0.97
Permanent Dentition	-0.31 (-0.68, 0.06) Stratum-specific p-value = 0.10	-0.35 (-0.71, 0.01) Stratum-specific p-value = 0.05	-0.04 (-0.22, 0.15) Stratum-specific p-value = 0.68	0.05 (-0.21, 0.32) Stratum-specific p-value = 0.68

*Adjusted for sex, age, presence of diagnosed health conditions, and HFIAS categories. Significant at p < 0.05.

### Other analyses

Table 3 shows unadjusted and adjusted associations between SiC status and anthropometric measures for all dentition and by dentition stage. The reference group for this table is the non-SiC category. ZBMI was generally below standard deviation means in all dentitions but not statistically significant. In our adjusted model HAZ, we found there was a statistically significant negative association with permanent dentition. After adjusting for sex, age, presence of diagnosed health conditions, and HFIAS categories, HAZ was on average 2.6 standard deviations below the mean (p=0.04).

**Table 3:** Association between SiC* and anthropometric measures (all dentition and interaction by dentition stage)**

	Height for age z-scores (HAZ) (95% CI)		Body mass index z-scores (ZBMI) (95% CI)	
	Unadjusted (N = 53)	Adjusted (N = 52)	Unadjusted (N = 53)	Adjusted (N = 52)
All Dentition	-0.05 (-0.60, 0.49) p-value = 0.85	0.01 (-0.56, 0.57) p-value = 0.99	-0.36 (-0.86, 0.13) p-value = 0.15	-0.33 (-0.81, 0.16) p-value = 0.18
Primary Dentition	0.83 (-0.26, 1.92) Stratum-specific p-value = 0.33	1.12 (-0.15, 2.38) Stratum-specific p-value = 0.08	0.09 (-1.24, 1.41) Stratum-specific p-value = 0.90	-0.09 (-1.60, 1.42) Stratum-specific p-value = 0.90
Mixed Dentition	0.08 (-0.51, 0.68) Stratum-specific p-value = 0.78	0.21 (-0.42, 0.84) Stratum-specific p-value = 0.50	-0.22 (-0.96, 0.53) Stratum-specific p-value = 0.56	-0.19 (-0.94, 0.55) Stratum-specific p-value = 0.61
Permanent Dentition	-2.31 (-5.22, 0.60) Stratum-specific p-value = 0.12	-2.64 (-5.20, -0.08)*** Stratum-specific p-value = 0.04	-1.67 (-3.61, 0.27) Stratum-specific p-value = 0.09	-1.21 (-3.69, 1.27) Stratum-specific p-value = 0.33

*SiC = mean dmft/DMFT of one third of the study group with the highest caries score

**Adjusted for sex, age, presence of diagnosed health conditions, and HFIAS categories

***Significant at p < 0.05. Stratum-specific p-value = 0.04.

## Discussion

### Key results

The purpose of our pilot study was to assess the association of dental caries with anthropometric deprivation in children residing in Claverito, a floating urban slum community in the Amazon. We hypothesized that anthropometric measurements of children with the greatest prevalence of caries would be impaired compared to children with lowest levels of caries. Despite our small sample size from our convenience sample of the community, results of this study were generally consistent with our hypothesis.

### Limitations

Our study had several limitations. First, our small sample size, which was restricted by our convenience sampling strategy of the community, limited our ability to detect statistically significant differences among other anthropometric measures. We were unable to obtain anthropometric data for 14 participants due to their inability to tolerate the measurements, which likely reduced the number of younger subjects with primary or mixed dentition. With a larger sample of participants in primary and mixed dentition, we may have detected significant assocations between SiC and anthropometric measures. Additionally, because of the small number of participants in the underweight and stunting groups, we were unable to analyze differences between SiC and non-SiC individuals stratified by anthropometric category.

Our study may not have been adequately powered to find a significant association of caries experience with continuous HAZ and ZBMI. Because nearly all participants had caries, those findings also may not have been significant. Since the SiC Index distinguishes individuals who are most severely affected by dental caries, our finding of an inverse linear relationship between dental disease reflected by SiC and HAZ is logical.

Existing research indicates that malnutrition is highly associated with poverty,^18^ and socioeconomic inequality in malnutrition exists throughout the world. Those with higher socioeconomic status have less malnutrition, and the resulting inequality is stronger for stunting than wasting.^19^ This is consistent with our finding of a significant association between SiC Index and height-for-age *z*-scores - a measure of stunting. Stunting is more prevalent than wasting: there are 144 million children under age 5 who suffer from stunting, while a third of that number suffer from wasting.^2^ Among our 67 participants, 13 were in the category of stunting compared to 1 in wasting. With a larger sample size, we may have detected a negative linear relationship between SiC Index and body mass index for age *z*-scores.

Another potential limitation relates to assessment of confounding factors. Childhood malnutrition is a complex situation mediated by a number of factors which confound the relation to dental caries. In our collaborative study, we were limited by what non-dental data was collected. For example, we had partial assessment of health status. Because access to medical care is limited in Claverito, participants might have had an undiagnosed health condition affecting their growth. Yet, we were able to collect basic information by proxy. Additionally, we were able to control for key demographic and socioeconomic variables used as covariates in our review of the literature, with Hoursehold Food Security Access Scale (HFIAS) used as a proxy for parental educational level.

Finally, as a cross-sectional study, we could only provide a snapshot of the association between oral disease and anthropometric deprivation. Longitudinal assessments are needed to provide full view of the impact of oral disease on children’s growth.

### Interpretation

While presence of caries was not associated with anthropometric deprivation, SiC was associated with height-for-age z-scores (HAZ). Because virtually all participants had caries, caries presence was not a sensitive enough indicator to demonstrate significant relationships with anthropometric deprivation. However, SiC identified those with the greatest number of caries and reflected disease severity. When adjustments were made for confounders, participants in the top third of number of caries of permanent teeth (SiC) had significantly lower height-for age z-scores (HAZ). It is possible that the cumulative effect of untreated caries affected height and that in our small sample size it was most evident among older children. Based on our results, there is evidence to suggest a relationship between dental disease and anthropometric deprivation. In the complex mosaic of resources that any given child needs for healthy development and growth, oral health is often overlooked.

 Peru has high prevalence of childhood caries (75.9% for children ages 6-7 years old and 91.2% for ages 11-12). This has been attributed to factors including socioeconomic disparities, health system fragmentation, insurance, limited provision of dental services, provider shortage, region, and caregiver educational level.^20^ Nationally, the mean DMFT score among 12-year-old children was reported to be 3.7.^21^ Claverito has an even greater prevalence of childhood caries (97%) and level of disease (mean dmft/DMFT of 7.2, SD: 4.7). Only 14 children in the study sample (21%) demonstrated access to care through clinical findings of dental fillings or extractions. Throughout the country, children residing in jungle regions have the most limited access to dental care. Only 21% of children in the jungle have access, compared with 39% residing in the urban Lima Metropolitan area.^20^ This was consistent with our study findings. Claverito’s unique geographic characteristics, coupled with socioeconomic factors limiting access, comprise a set of challenges faced by other communities that need to be addressed to promote oral and systemic health.

There are some inconsistent findings in the literature on the relationship between dental caries and pediatric anthropometric measures. Factors attributed to this inconsistency include uncontrolled confounders, sampling bias, participant ages, methods of categorization of anthropometrics, and varying definitions of caries.^7^ ^),(^ ^22^ In our study, anthropometrics served as a proxy of systemic health. This study expands on the existing literature by exploring the relationship between oral and systemic health specifically on potential impacts on the growth of children residing in a floating slum community, facing poverty, and experiencing extremely high prevalence of dental disease.

Regional findings from other low-income, under-resourced communities are consistent with our results. Negative impacts of oral disease on 1407 children from birth to age 6 in an indigenous, geographically-remote population in the Ecuadorian Amazon included sleep disturbances and poor nutritional status.^6^ Similar findings were reported earlier in the region, where a community-based oral health and nutrition intervention was implemented among 1575 children from birth to age 6. That study found a significant reduction in dental caries and caries-related malnutrition among the intervention group,^23^ which lends support to a causal relationship between dental disease and adverse childhood growth outcomes.

### Generalizability

Our study contributes to the growing body of evidence on a relationship between dental disease and anthropometric deprivation in underserved communities. After adjusting for confounding variables, we found an inverse relationship between the highest levels of caries and stunting, as measured by height-for-age *z* scores.

Peru has high prevalence of childhood caries (75.9% for children ages 6-7 years old and 91.2% for ages 11-12), and the mean DMFT score among 12-year-old children is 3.7.^21^ Claverito has an even greater prevalence of childhood caries (97%) and level of disease (mean dmft/DMFT of 7.2).

Globally, oral health in communities with a high incidence of poverty are under-resourced. An estimated 249 million children in low- and middle-income countries are at risk of not reaching their developmental potential due to stunting or extreme poverty.^24^ Childhood growth and development is multifactorial, and oral health is an often-overlooked component. However, dental caries is the most common chronic childhood disease and has significant impacts on systemic health and quality of life. Understanding and addressing barriers to oral health care is a critical component of an integrated response to the World Health Organization’s Sustainable Development Goals.
